# Supplementary material for: Lumpy skin disease outbreaks in Egypt during 2017-2018 among sheeppox vaccinated cattle: Epidemiological, pathological, and molecular findings
Source: PLoS One. 2021 Oct 20;16(10):e0258755. doi: 10.1371/journal.pone.0258755 (PMC8528327; doi:10.1371/journal.pone.0258755)
Supplement: S1 Raw images — (PDF) [file pone.0258755.s001.pdf]

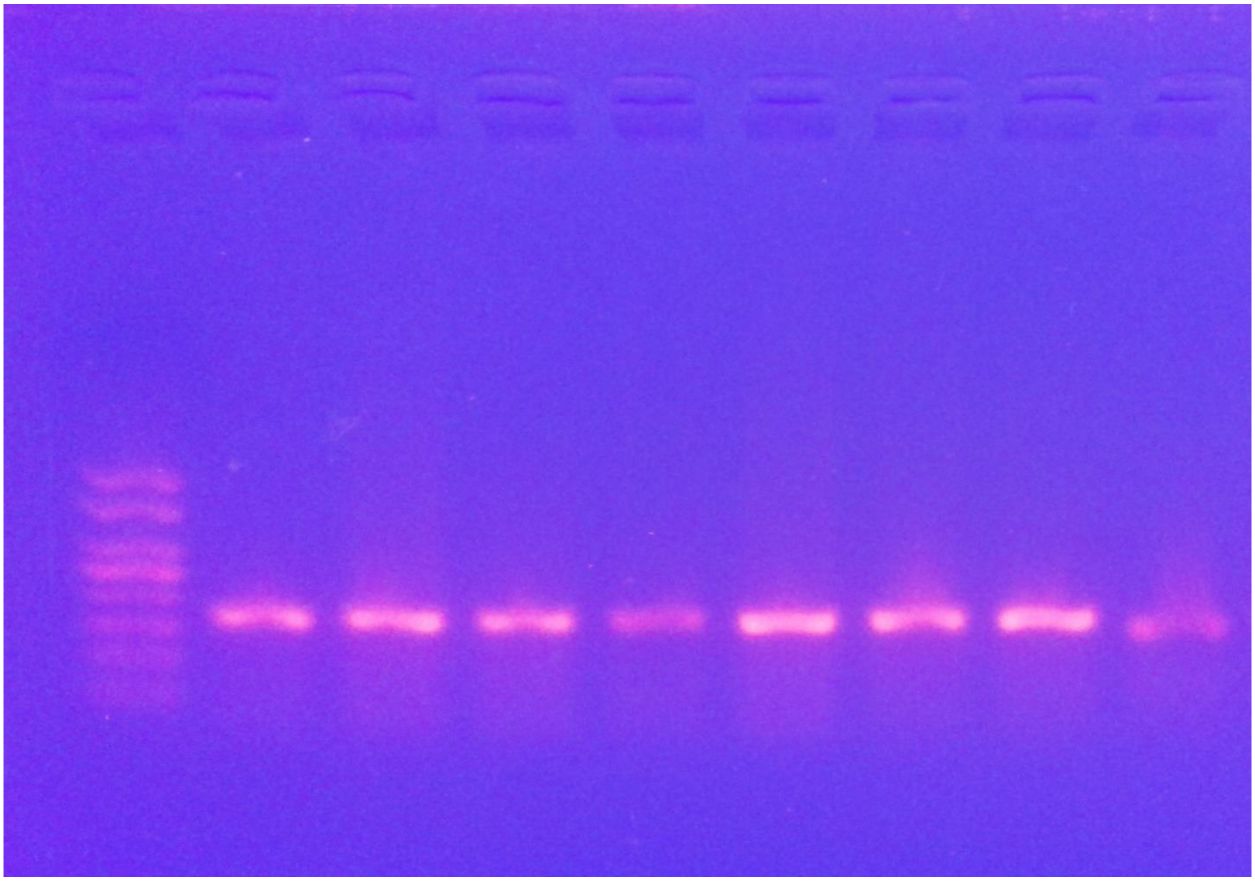

Original image for fig.3

Fig. 3. Gel electrophoresis of PCR products

Gel electrophoresis of PCR products using RPO30 specific primer set using 50bp ladder (with 172 bp expected product for LSDV (Lane 2 to lane 8) and 151 for SPPV (Lane: 9).
